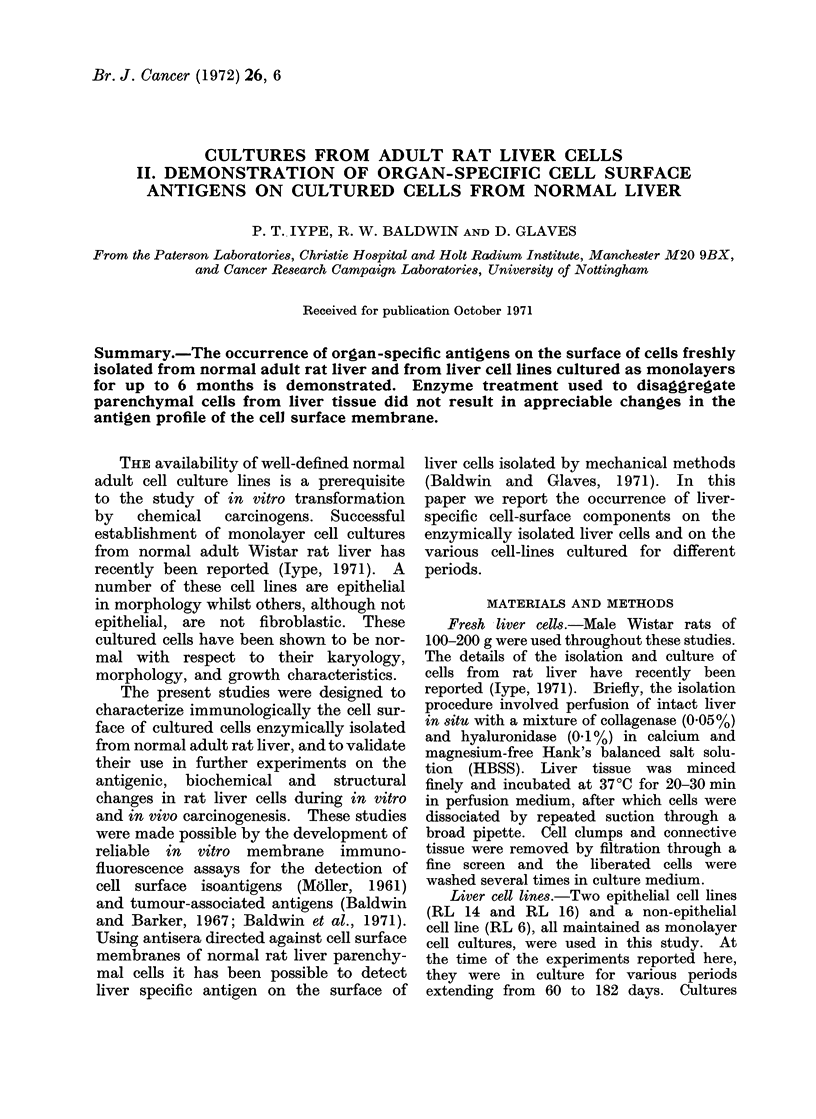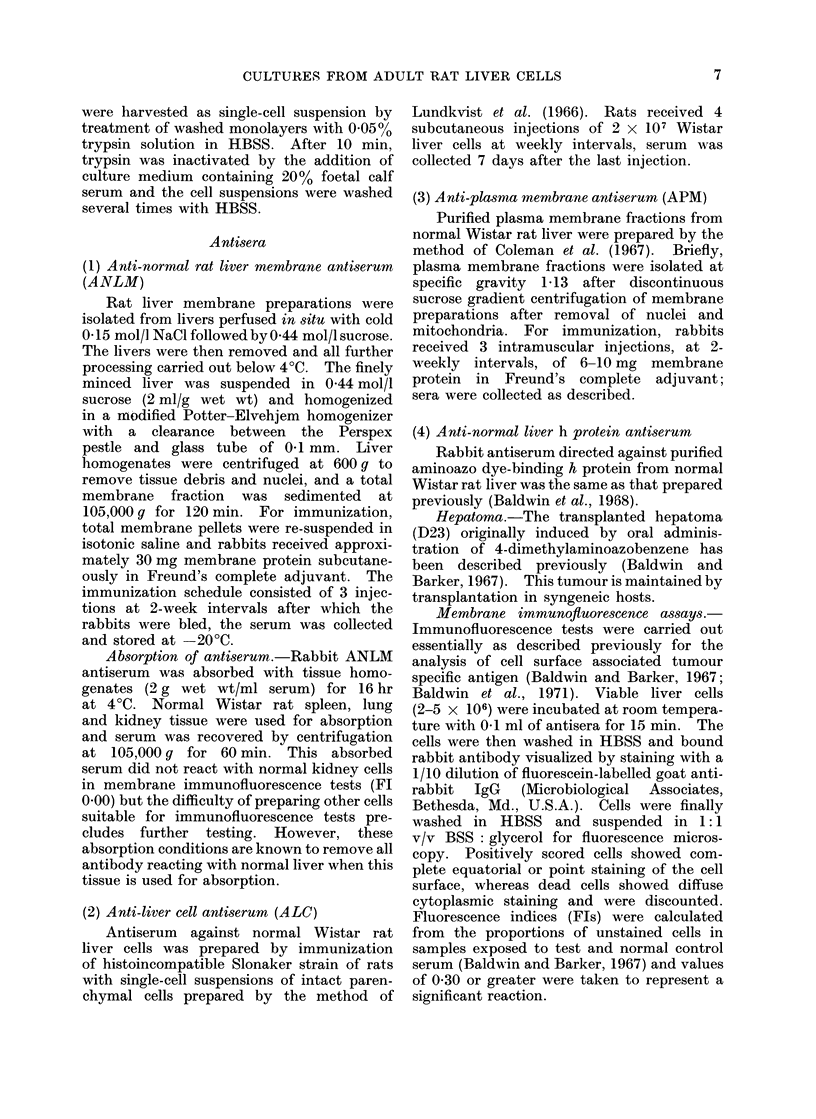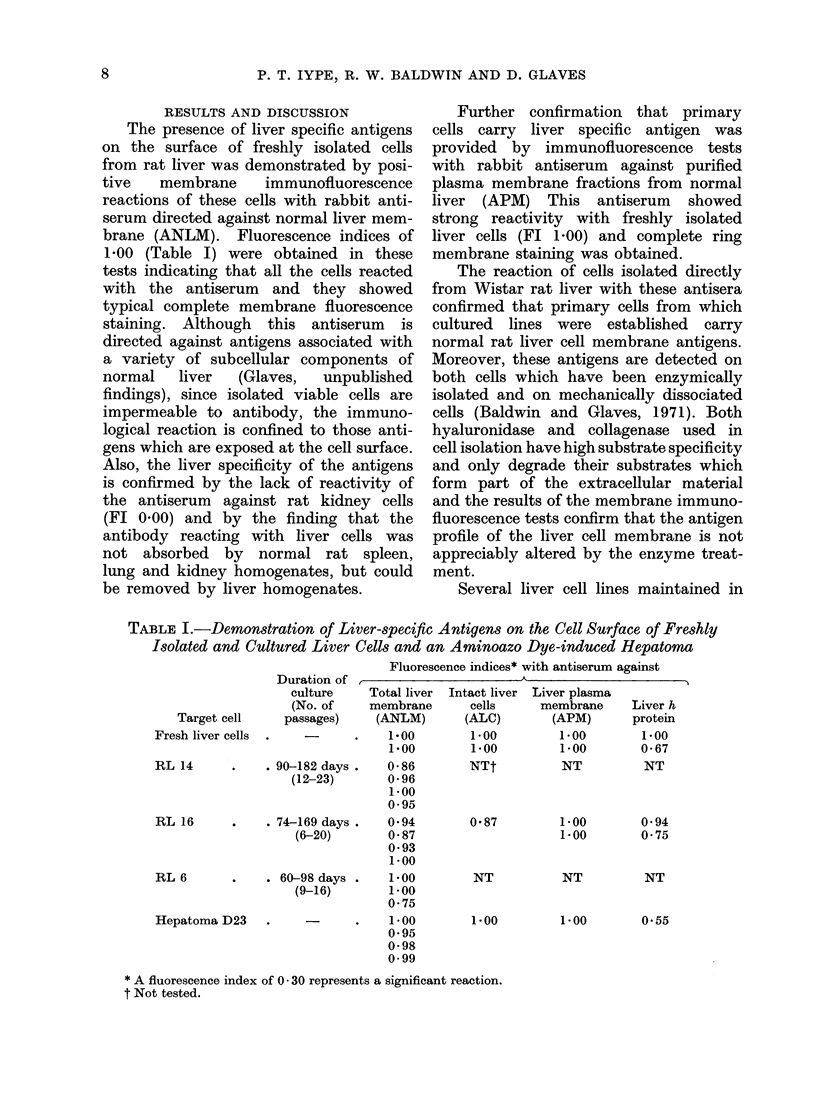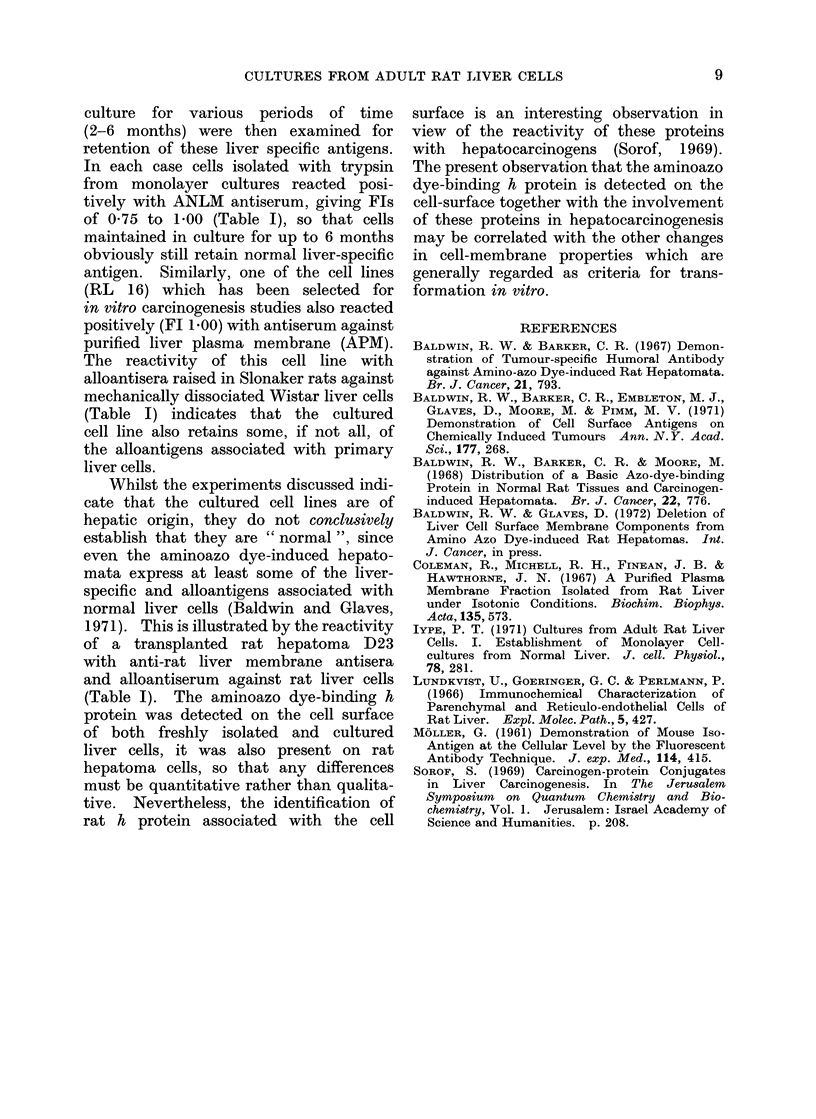# Cultures from Adult Rat Liver Cells. II. Demonstration of Organ-Specific Cell Surface Antigens on Cultured Cells from Normal Liver

**DOI:** 10.1038/bjc.1972.3

**Published:** 1972-02

**Authors:** P. T. Iype, R. W. Baldwin, D. Glaves

## Abstract

The occurrence of organ-specific antigens on the surface of cells freshly isolated from normal adult rat liver and from liver cell lines cultured as monolayers for up to 6 months is demonstrated. Enzyme treatment used to disaggregate parenchymal cells from liver tissue did not result in appreciable changes in the antigen profile of the cell surface membrane.


*Br. J. Cancer* (1972) **26**, 6

### CULTURES FROM ADULT RAT LIVER CELLS II. DEMONSTRATION OF ORGAN-SPECIFIC CELL SURFACE ANTIGENS ON CULTURED CELLS FROM NORMAL LIVER

P. T. IYPE, R. W. BALDWIN AND D. GLAVES

*From the Paterson Laboratories, Christie Hospital and Holt Radium Institute, Manchester M20 9BX, and Cancer Research Campaign Laboratories, University of Nottingham*

Received for publication October 1971

**Summary.—The occurrence of organ-specific antigens on the surface of cells freshly isolated from normal adult rat liver and from liver cell lines cultured as monolayers for up to 6 months is demonstrated. Enzyme treatment used to disaggregate parenchymal cells from liver tissue did not result in appreciable changes in the antigen profile of the cell surface membrane.**

THE availability of well-defined normal adult cell culture lines is a prerequisite to the study of *in vitro* transformation by chemical carcinogens. Successful establishment of monolayer cell cultures from normal adult Wistar rat liver has recently been reported (Iype, 1971). A number of these cell lines are epithelial in morphology whilst others, although not epithelial, are not fibroblastic. These cultured cells have been shown to be normal with respect to their karyology, morphology, and growth characteristics.

The present studies were designed to characterize immunologically the cell surface of cultured cells enzymically isolated from normal adult rat liver, and to validate their use in further experiments on the antigenic, biochemical and structural changes in rat liver cells during *in vitro* and *in vivo* carcinogenesis. These studies were made possible by the development of reliable *in vitro* membrane immunofluorescence assays for the detection of cell surface isoantigens (Möller, 1961) and tumour-associated antigens (Baldwin and Barker, 1967; Baldwin *et al.*, 1971). Using antisera directed against cell surface membranes of normal rat liver parenchymal cells it has been possible to detect liver specific antigen on the surface of liver cells isolated by mechanical methods (Baldwin and Glaves, 1971). In this paper we report the occurrence of liver-specific cell-surface components on the enzymically isolated liver cells and on the various cell-lines cultured for different periods.

### MATERIALS AND METHODS

*Fresh liver cells.*—Male Wistar rats of 100–200 g were used throughout these studies. The details of the isolation and culture of cells from rat liver have recently been reported (Iype, 1971). Briefly, the isolation procedure involved perfusion of intact liver *in situ* with a mixture of collagenase (0·05%) and hyaluronidase (0·1%) in calcium and magnesium-free Hank's balanced salt solution (HBSS). Liver tissue was minced finely and incubated at 37°C for 20–30 min in perfusion medium, after which cells were dissociated by repeated suction through a broad pipette. Cell clumps and connective tissue were removed by filtration through a fine screen and the liberated cells were washed several times in culture medium.

*Liver cell lines.*—Two epithelial cell lines (RL 14 and RL 16) and a non-epithelial cell line (RL 6), all maintained as monolayer cell cultures, were used in this study. At the time of the experiments reported here, they were in culture for various periods extending from 60 to 182 days. Cultures were harvested as single-cell suspension by treatment of washed monolayers with 0·05% trypsin solution in HBSS. After 10 min, trypsin was inactivated by the addition of culture medium containing 20% foetal calf serum and the cell suspensions were washed several times with HBSS.

### *Antisera*

(1) *Anti-normal rat liver membrane antiserum* (*ANLM*)

Rat liver membrane preparations were isolated from livers perfused *in situ* with cold 0·15 mol/l NaCl followed by 0·44 mol/l sucrose. The livers were then removed and all further processing carried out below 4 °C. The finely minced liver was suspended in 0·44 mol/l sucrose (2 ml/g wet wt) and homogenized in a modified Potter–Elvehjem homogenizer with a clearance between the Perspex pestle and glass tube of 0·1 mm. Liver homogenates were centrifuged at 600 *g* to remove tissue debris and nuclei, and a total membrane fraction was sedimented at 105,000 *g* for 120 min. For immunization, total membrane pellets were re-suspended in isotonic saline and rabbits received approximately 30 mg membrane protein subcutaneously in Freund's complete adjuvant. The immunization schedule consisted of 3 injections at 2-week intervals after which the rabbits were bled, the serum was collected and stored at −20 °C.

*Absorption of antiserum.*—Rabbit ANLM antiserum was absorbed with tissue homogenates (2 g wet wt/ml serum) for 16 hr at 4 °C. Normal Wistar rat spleen, lung and kidney tissue were used for absorption and serum was recovered by centrifugation at 105,000 *g* for 60 min. This absorbed serum did not react with normal kidney cells in membrane immunofluorescence tests (FI 0·00) but the difficulty of preparing other cells suitable for immunofluorescence tests precludes further testing. However, these absorption conditions are known to remove all antibody reacting with normal liver when this tissue is used for absorption.

(2) *Anti-liver cell antiserum* (*ALC*)

Antiserum against normal Wistar rat liver cells was prepared by immunization of histoincompatible Slonaker strain of rats with single-cell suspensions of intact parenchymal cells prepared by the method of Lundkvist *et al.* (1966). Rats received 4 subcutaneous injections of $2 \times 10^7$ Wistar liver cells at weekly intervals, serum was collected 7 days after the last injection.

(3) *Anti-plasma membrane antiserum* (APM)

Purified plasma membrane fractions from normal Wistar rat liver were prepared by the method of Coleman *et al.* (1967). Briefly, plasma membrane fractions were isolated at specific gravity 1·13 after discontinuous sucrose gradient centrifugation of membrane preparations after removal of nuclei and mitochondria. For immunization, rabbits received 3 intramuscular injections, at 2-weekly intervals, of 6–10 mg membrane protein in Freund's complete adjuvant; sera were collected as described.

(4) *Anti-normal liver* h *protein antiserum*

Rabbit antiserum directed against purified aminoazo dye-binding *h* protein from normal Wistar rat liver was the same as that prepared previously (Baldwin *et al.*, 1968).

*Hepatoma.*—The transplanted hepatoma (D23) originally induced by oral administration of 4-dimethylaminoazobenzene has been described previously (Baldwin and Barker, 1967). This tumour is maintained by transplantation in syngeneic hosts.

*Membrane immunofluorescence assays.*—Immunofluorescence tests were carried out essentially as described previously for the analysis of cell surface associated tumour specific antigen (Baldwin and Barker, 1967; Baldwin *et al.*, 1971). Viable liver cells ($2–5 \times 10^6$) were incubated at room temperature with 0·1 ml of antisera for 15 min. The cells were then washed in HBSS and bound rabbit antibody visualized by staining with a 1/10 dilution of fluorescein-labelled goat anti-rabbit IgG (Microbiological Associates, Bethesda, Md., U.S.A.). Cells were finally washed in HBSS and suspended in 1 : 1 v/v BSS : glycerol for fluorescence microscopy. Positively scored cells showed complete equatorial or point staining of the cell surface, whereas dead cells showed diffuse cytoplasmic staining and were discounted. Fluorescence indices (FIs) were calculated from the proportions of unstained cells in samples exposed to test and normal control serum (Baldwin and Barker, 1967) and values of 0·30 or greater were taken to represent a significant reaction.

### RESULTS AND DISCUSSION

The presence of liver specific antigens on the surface of freshly isolated cells from rat liver was demonstrated by positive membrane immunofluorescence reactions of these cells with rabbit antiserum directed against normal liver membrane (ANLM). Fluorescence indices of 1·00 (Table I) were obtained in these tests indicating that all the cells reacted with the antiserum and they showed typical complete membrane fluorescence staining. Although this antiserum is directed against antigens associated with a variety of subcellular components of normal liver (Glaves, unpublished findings), since isolated viable cells are impermeable to antibody, the immunological reaction is confined to those antigens which are exposed at the cell surface. Also, the liver specificity of the antigens is confirmed by the lack of reactivity of the antiserum against rat kidney cells (FI 0·00) and by the finding that the antibody reacting with liver cells was not absorbed by normal rat spleen, lung and kidney homogenates, but could be removed by liver homogenates.

Further confirmation that primary cells carry liver specific antigen was provided by immunofluorescence tests with rabbit antiserum against purified plasma membrane fractions from normal liver (APM) This antiserum showed strong reactivity with freshly isolated liver cells (FI 1·00) and complete ring membrane staining was obtained.

The reaction of cells isolated directly from Wistar rat liver with these antisera confirmed that primary cells from which cultured lines were established carry normal rat liver cell membrane antigens. Moreover, these antigens are detected on both cells which have been enzymically isolated and on mechanically dissociated cells (Baldwin and Glaves, 1971). Both hyaluronidase and collagenase used in cell isolation have high substrate specificity and only degrade their substrates which form part of the extracellular material and the results of the membrane immunofluorescence tests confirm that the antigen profile of the liver cell membrane is not appreciably altered by the enzyme treatment.

Several liver cell lines maintained in

TABLE I.—*Demonstration of Liver-specific Antigens on the Cell Surface of Freshly Isolated and Cultured Liver Cells and an Aminoazo Dye-induced Hepatoma*

| Target cell | Duration of culture (No. of passages) | Fluorescence indices* with antiserum against | | | |
|---|---|---|---|---|---|
| | | Total liver membrane (ANLM) | Intact liver cells (ALC) | Liver plasma membrane (APM) | Liver *h* protein |
| Fresh liver cells | — | 1·00<br>1·00 | 1·00<br>1·00 | 1·00<br>1·00 | 1·00<br>0·67 |
| RL 14 | 90–182 days (12–23) | 0·86<br>0·96<br>1·00<br>0·95 | NT† | NT | NT |
| RL 16 | 74–169 days (6–20) | 0·94<br>0·87<br>0·93<br>1·00 | 0·87 | 1·00<br>1·00 | 0·94<br>0·75 |
| RL 6 | 60–98 days (9–16) | 1·00<br>1·00<br>0·75 | NT | NT | NT |
| Hepatoma D23 | — | 1·00<br>0·95<br>0·98<br>0·99 | 1·00 | 1·00 | 0·55 |

* A fluorescence index of 0·30 represents a significant reaction.
† Not tested.

culture for various periods of time (2–6 months) were then examined for retention of these liver specific antigens. In each case cells isolated with trypsin from monolayer cultures reacted positively with ANLM antiserum, giving FIs of 0·75 to 1·00 (Table I), so that cells maintained in culture for up to 6 months obviously still retain normal liver-specific antigen. Similarly, one of the cell lines (RL 16) which has been selected for *in vitro* carcinogenesis studies also reacted positively (FI 1·00) with antiserum against purified liver plasma membrane (APM). The reactivity of this cell line with alloantisera raised in Slonaker rats against mechanically dissociated Wistar liver cells (Table I) indicates that the cultured cell line also retains some, if not all, of the alloantigens associated with primary liver cells.

Whilst the experiments discussed indicate that the cultured cell lines are of hepatic origin, they do not *conclusively* establish that they are " normal ", since even the aminoazo dye-induced hepatomata express at least some of the liver-specific and alloantigens associated with normal liver cells (Baldwin and Glaves, 1971). This is illustrated by the reactivity of a transplanted rat hepatoma D23 with anti-rat liver membrane antisera and alloantiserum against rat liver cells (Table I). The aminoazo dye-binding *h* protein was detected on the cell surface of both freshly isolated and cultured liver cells, it was also present on rat hepatoma cells, so that any differences must be quantitative rather than qualitative. Nevertheless, the identification of rat *h* protein associated with the cell surface is an interesting observation in view of the reactivity of these proteins with hepatocarcinogens (Sorof, 1969). The present observation that the aminoazo dye-binding *h* protein is detected on the cell-surface together with the involvement of these proteins in hepatocarcinogenesis may be correlated with the other changes in cell-membrane properties which are generally regarded as criteria for transformation *in vitro*.

### REFERENCES

Baldwin, R. W. & Barker, C. R. (1967) Demonstration of Tumour-specific Humoral Antibody against Amino-azo Dye-induced Rat Hepatomata. *Br. J. Cancer*, **21**, 793.

Baldwin, R. W., Barker, C. R., Embleton, M. J., Glaves, D., Moore, M. & Pimm, M. V. (1971) Demonstration of Cell Surface Antigens on Chemically Induced Tumours *Ann. N.Y. Acad. Sci.*, **177**, 268.

Baldwin, R. W., Barker, C. R. & Moore, M. (1968) Distribution of a Basic Azo-dye-binding Protein in Normal Rat Tissues and Carcinogen-induced Hepatomata. *Br. J. Cancer*, **22**, 776.

Baldwin, R. W. & Glaves, D. (1972) Deletion of Liver Cell Surface Membrane Components from Amino Azo Dye-induced Rat Hepatomas. *Int. J. Cancer*, in press.

Coleman, R., Michell, R. H., Finean, J. B. & Hawthorne, J. N. (1967) A Purified Plasma Membrane Fraction Isolated from Rat Liver under Isotonic Conditions. *Biochim. Biophys. Acta*, **135**, 573.

Iype, P. T. (1971) Cultures from Adult Rat Liver Cells. I. Establishment of Monolayer Cell-cultures from Normal Liver. *J. cell. Physiol.*, **78**, 281.

Lundkvist, U., Goeringer, G. C. & Perlmann, P. (1966) Immunochemical Characterization of Parenchymal and Reticulo-endothelial Cells of Rat Liver. *Expl. Molec. Path.*, **5**, 427.

Möller, G. (1961) Demonstration of Mouse Iso-Antigen at the Cellular Level by the Fluorescent Antibody Technique. *J. exp. Med.*, **114**, 415.

Sorof, S. (1969) Carcinogen-protein Conjugates in Liver Carcinogenesis. In *The Jerusalem Symposium on Quantum Chemistry and Biochemistry*, Vol. 1. Jerusalem: Israel Academy of Science and Humanities. p. 208.